# Dynamic Buckling Analysis of Thin Film/Polydimethylsiloxane Substrate Structures in Curved State with Finite Thickness

**DOI:** 10.3390/polym18030411

**Published:** 2026-02-05

**Authors:** Haohao Bi, Wenjie Li, Liuyun Wang

**Affiliations:** School of Science, Qingdao University of Technology, Qingdao 266520, China; 15763049211@163.com (W.L.); 15281673561@163.com (L.W.)

**Keywords:** thin film/substrate structure, energy minimization, chaos

## Abstract

Curved sensors hold significant positions in various fields of modern science and technology, such as medical care, soft robotics, and electronic devices. Meanwhile, flexible electronic devices with film/polydimethylsiloxane substrate structures have been widely applied in the configuration design and performance enhancement of sensors. It is essential to consider the dynamic buckling behavior of film/substrate structures under bending conditions for the optimization of sensor functions. In this study, the dynamic behaviors of thin film/substrate structures with finite thickness in the curved state are investigated. Firstly, the dynamic equations considering damping and external excitation are established based on the principle of minimum energy and the Lagrange function. Secondly, the dynamic responses under different parameters are analyzed. Finally, the effects of the frequency of external excitation, pre-strain, the amplitude of external excitation strain, and the Young’s modulus and thickness of the substrate on the critical value of chaos occurrence are discussed respectively. This study is aimed at providing novel insights for the design of curved sensors based on thin film/substrate structures.

## 1. Introduction

With the advancement in frontier scientific and technological fields such as materials science, flexible sensors, owing to their superior bendability, stretchability, and stability, have been extensively applied in frontier domains, including wearable electronic devices and intelligent robotics [1,2,3,4,5,6]. Wrinkle-type sensors based on the film/substrate structure play a significant role. Under operating conditions, different dynamic modes may occur in the film/substrate structure, thereby affecting the working performance of the sensors. Therefore, it is of great significance to analyze the buckling behavior of the film/substrate structure under bending conditions for both the improvement of the performance of bending sensors and the optimization of their design.

Over the past few years, flexible bending sensors have been widely applied in soft robotics technology and electronic wearable devices, and it is noteworthy that they have attracted considerable attention in both theoretical and engineering fields. To enable these sensors to better serve the field of science and technology, significant efforts have been devoted by numerous scholars to the structural improvement and performance optimization of bending sensors. Lee et al. [7] investigated the various characteristics of flexible bending sensors with an inverted pyramid structure, which enhanced the dynamic range of bending strain of the sensors to 0.025–5.4%. A bending sensor, which is applicable for measuring the bending angle of soft robotic fingers and features ease of integration, together with an effective sensing technique, was proposed by Kar et al. [8], thereby facilitating the realization of repeatable characteristics. With respect to bending sensors based on different structures, aiming at the issue of difficulty in perceiving bending directions and stresses, corresponding theoretical or experimental studies have been conducted by researchers. For crack-based sensors, crack spacing, crack direction, crack generation mechanism, and the relationship between the bending axis of the film and the crack direction have been investigated by Kwon et al. [9]. To enhance mechanical properties, Yan et al. [10] proposed a novel integrated piezoelectric bending sensor, which exhibits a tensile strength of at least 51 MPa, a shear strength of 28 MPa, and an interfacial toughness of 300 J/m^2^. The performance of flexible sensors is primarily determined by the selection of sensitive materials and the structural design of devices [11]. To further enhance the sensitivity of flexible sensors, different shapes and structures of flexible sensors have been researched and designed by researchers [12,13,14,15,16,17]. Among them, optimized sensing devices based on surface wrinkling can simultaneously exhibit high deformability and excellent sensing performance, and film/substrate structures with wrinkled configurations can be employed for the design of flexible sensors [18]. For flexible sensors based on wrinkled structures, substantially important studies have been carried out by numerous researchers in both theoretical and experimental aspects. Park et al. [19] introduced wrinkles into carbon nanotube films and coupled two wrinkled electrodes, which improved the pressure sensitivity by 12,800 times and reduced the response time to within 20 ms. For flexible piezoelectric capacitive sensors based on wrinkled microstructures, Baek et al. [20] compared wrinkle-free, single-sided wrinkled, and double-sided wrinkled films, and found that the response time and release time of the double-sided wrinkled pressure sensor were improved by 42% and 25%, respectively. And Tang et al. [21] developed a piezoresistive sensor by combining controllable graphene nanowall wrinkles with a polydimethylsiloxane (PDMS) substrate, which features a fast response speed within 6.9 ms and a low limit of detection (LOD) of 2 mg. To improve the mechanical stability and stretchability of sensors, Hu et al. [22] proposed a strain sensor, which achieves a maximum strain of 75% and a gauge factor of 6657 by combining a stretchable wrinkled structure with a high-sensitivity microcrack sensor. Liu et al. [23] proposed a flexible piezoresistive sensor composed of silver nanowire-coated polydimethylsiloxane (PDMS) based on a gradient wrinkled structure, which achieves a detection range of 10–50 kPa. In addition, Lei et al. [24] proposed a pressure sensor that adopts a gradient wrinkled electrospun polyurethane membrane, achieving a wide sensing range of 0–260 kPa. Based on nested wrinkle/crack microstructures, Yan et al. [25] proposed a high-sensitivity strain sensor, which achieves an amplification factor of up to 7805.9, stretchability over a strain range of 60%, and stability under 1000 stretch-release cycles. Based on a hierarchical wrinkle/crack structure, Yuan et al. [26] realized three sensing characteristics in a single pressure sensor, where the CD exhibits good linearity above 20 MPa. Given the important role of wrinkled structures in sensor design, it is, therefore, plausible that the dynamic buckling of film/substrate structures relying on the mechanical buckling principle may exert an impact on the stability and other performances of sensors.

Furthermore, during the operation of film/substrate structures with wrinkled structures, different dynamic behaviors may be exhibited depending on variations in pre-strain or stress. It is essential that the investigation of their dynamic buckling vibration be conducted for the performance optimization of wrinkled sensors. In terms of the theoretical research and application of film/substrate structures under bending conditions, based on statics, the energy minimization method was applied by Ma et al. [27] for static modeling of film/substrate structures in the bent state, which was found to be consistent with the finite element results. Building on this foundation, a flexible sensor based on the film/substrate structure was applied to capsule endoscopy by Bi et al. [28], and this sensor was employed to detect the contact pressure between the inner wall of the small intestine and the vibration-impact capsule robot. In the field of dynamics, Bi et al. [29] investigated the influence of pre-strain and the Young’s modulus of the substrate on the dynamic response of energy harvesters, which facilitates the optimization of energy harvesters based on buckled piezoelectric ribbon/substrate structures. Regarding the issue of three-layer film/substrate structures, Wang et al. [30] explored the effects of the parameters of the intermediate layer on the nonlinear frequencies of corrugated three-layer structures. Furthermore, Bi et al. [31] studied the influence of the intermediate layer on the wrinkling instability of three-layer structures. To address the performance issues of piezoelectric nanostructures, Wang et al. [32] studied the wrinkling problem of film/substrate structures, and the influence of voltage on the surface wrinkling of elastomers was revealed. In order to reveal the influence of layered nanofilms on the dynamic behaviors of substrates, Dong et al. [33] studied the effect of sub-nanoscale van der Waals (vdWs) dynamic boundaries on the dynamic buckling of film/substrate structures. Considering that wearable electronic devices operate in complex vibration environments, to ensure the stability of substrate structures in dynamic environments, Bi et al. [34] investigated the influence of Gaussian white noise on the dynamic response of film/substrate structures. Building on the important studies conducted, the buckled structures under bending conditions will be further considered.

With the extensive adoption of wearable sensors, it is particularly crucial to investigate film/substrate structures under bending conditions. Building on the previous research, the dynamic buckling behavior of film/substrate structures subjected to periodically excited strain under bending conditions is investigated in this work. Compared with the previous research on the dynamic behavior of thin film/substrate structure, this paper considers the bending configuration in order to better fit the actual structure of the bending sensor, which is more in line with the actual situation of the bending sensor at work. In Section 2 of the paper, the vibration control equation of film/substrate structures with periodic excitation and damping is derived by the principle of minimum energy and the Lagrange function method. In Section 3, numerical simulations are performed on the control equation to explore the effects of the pre-strain, the Young’s modulus and thickness of the substrate, the frequency of the external excitation strain, and the amplitude of the excited strain on the stability and vibration modes, respectively. In Section 4, conclusions are presented.

## 2. Materials and Methods

**Figure 1:**
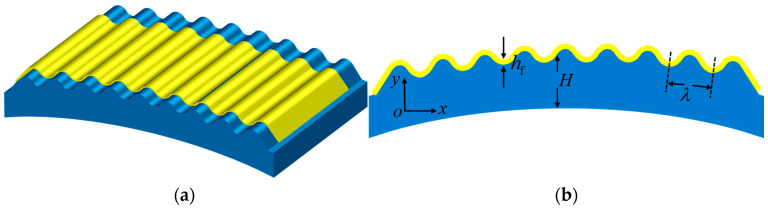
(**a**) Schematic diagram of the thin film/substrate structure under the bending state; (**b**) front view of the thin film/substrate structure under the bending state.

In the present study, the thin film/substrate structure with damping and periodic external excitation strain under the bending state is considered, which is constituted by the thin film undertaking the functional operations of electronic devices and the substrate with finite thickness. The thin film/substrate structure in the bending state is illustrated in Figure 1, where *h*_f_ denotes the thickness of the thin film, *H* represents that of the substrate, and *λ* stands for the wavelength.

According to Reference [28], the lateral displacement of the thin film, denoted as *w*(*x*)

(1) $$w\left(x\right)=A\cos\left(kx\right)$$

where *A* denotes the time-dependent wrinkling amplitude of the structure and $k=\frac{2\pi }{\lambda }$ is the characteristic wave number.

The central axial strain of the thin film can be expressed as

(2) $$\varepsilon_{x}=-\varepsilon +\frac{\partial u}{\partial x}+\frac{1}{2}{\left(\frac{\partial w}{\partial x}\right)}^{2},$$

where *ε* denotes the strain of the thin film and *u* denotes the axial displacement.

Due to

(3) $$\frac{\partial \varepsilon_{x}}{\partial x}=\frac{\partial^{2}u}{\partial x^{2}}+\frac{\partial w}{\partial x}\frac{\partial^{2}w}{\partial x^{2}}=0.$$

Then

(4) $$\left\{\begin{array}{l} w=A(t)\cos(kx)\\ u=\frac{1}{8}kA^{2}(t)\sin(2kx) \end{array}\right.,$$

According to References [27,35], the film energy *U*_m_ can be expressed as

(5) $$U_{m}=\frac{1}{2}{\bar{E}}_{f}h_{f}\frac{k}{2\pi }\int_{0}^{\frac{2\pi }{k}}{\left(-\varepsilon +\frac{\partial u}{\partial x}+\frac{1}{2}{\left(\frac{\partial w}{\partial x}\right)}^{2}\right)}^{2}dx,$$

where ${\bar{E}}_{f}=E_{f}/1-\nu_{f}^{2}$, *ν*_f_ denotes the Poisson’s ratio of the thin film and *E*_f_ denotes the Young’s modulus of the thin film. The bending energy of the thin film, denoted as *U*_b_, can be expressed as

(6) $$U_{b}=\frac{1}{2}{\bar{E}}_{f}\frac{k}{2\pi }\int_{0}^{\frac{2\pi }{k}}\int_{\frac{-h_{f}}{2}}^{\frac{h_{f}}{2}}{\left(\frac{\partial^{2}w}{\partial x^{2}}y\right)}^{2}dydx.$$

It can be known from References [27,28,35] that the energy of the substrate, denoted as *U*_s_, is

(7) $$U_{s}=\frac{k}{2\pi }\int_{0}^{\frac{2\pi }{k}}\frac{1}{2}g(kH){\bar{E}}_{s}kw^{2}dx+\frac{1}{2}{\bar{E}}_{s}\int_{0}^{H}{\left[\varepsilon_{\mathrm{pre}}-\varepsilon -\kappa \left(H-y\right)\right]}^{2}dy,$$

where ${\bar{E}}_{s}=E_{s}/1-\nu_{s}^{2}$, $g\left(kH\right)=\frac{\left(3-4\nu_{s}\right)\cosh\left(2kH\right)+5-12\nu_{s}+8\nu_{s}^{2}+2{\left(kH\right)}^{2}}{\left(6-8\nu_{s}\right)\sinh\left(2kH\right)-4kH}$, *ν*_s_ denotes the Poisson’s ratio of the substrate, *E*_s_ represents the Young’s modulus of the substrate, *H* stands for the thickness of the substrate, and *κ* represents the curvature of the substrate. *ε*_pre_ denotes the pre-strain, and the pre-strain of this structure varies from 0% to 10% [36].

The film energy, denoted as *U*_m_, the bending energy, denoted as *U*_b_, and the substrate energy, denoted as *U*_s_, can be finally obtained.

(8) $$\left\{\begin{array}{ll} U_{b}= & \frac{1}{48}{\bar{E}}_{f}h_{f}^{3}k^{4}A^{2}\\ U_{m}= & \frac{1}{2}{\bar{E}}_{f}h_{f}\varepsilon^{2}+\frac{1}{32}{\bar{E}}_{f}h_{f}k^{4}A^{4}-\frac{1}{4}{\bar{E}}_{f}h_{f}k^{2}A^{2}\varepsilon \\ U_{s}= & \frac{1}{2}{\bar{E}}_{s}\int_{0}^{H}{\left[\varepsilon_{\mathrm{pre}}-\varepsilon -\kappa (H-y)\right]}^{2}dy+\frac{{\bar{E}}_{s}}{4}g(kH)kA^{2} \end{array}\right..$$

It should be indicated that the total energy, denoted as *U*_total_, can be formulated as *U*_total_ = *U*_b_ + *U*_m_ + *U*_s_; accordingly,

(9) $$\begin{array}{cc} U_{\mathrm{total}} & =\frac{1}{2}{\bar{E}}_{f}h_{f}\varepsilon^{2}+\frac{1}{32}{\bar{E}}_{f}h_{f}k^{4}A^{4}+\frac{1}{4}{\bar{E}}_{f}h_{f}k^{2}A^{2}\left(f-\varepsilon \right)\\ & \;+\frac{1}{2}{\bar{E}}_{s}\int_{0}^{H}{\left[\varepsilon_{\mathrm{pre}}-\varepsilon -\kappa \left(H-y\right)\right]}^{2}dy \end{array},$$

where

(10) $$f=\frac{h_{f}^{2}k^{2}}{12}+\frac{{\bar{E}}_{s}g\left(kH\right)}{{\bar{E}}_{f}kh_{f}}.$$

It is specified that a periodic excitation is incorporated into the substrate along its curvature.

(11) $$\begin{array}{cc} U_{\mathrm{total}} & =\frac{1}{2}{\bar{E}}_{f}h_{f}\varepsilon^{2}+\frac{1}{32}{\bar{E}}_{f}h_{f}k^{4}A^{4}+\frac{1}{4}{\bar{E}}_{f}h_{f}k^{2}A^{2}\left(f-\varepsilon \right)\\ & \;+\frac{1}{2}{\bar{E}}_{s}\int_{0}^{H}{\left[\varepsilon_{\mathrm{pre}}-\varepsilon +\varepsilon_{\mathrm{applied}}\cos(2\pi \omega t)-\kappa \left(H-y\right)\right]}^{2}dy \end{array},$$

where *ε*_applied_ denotes the amplitude of the external excitation strain and *ω* represents the frequency of the external excitation strain.

Minimizing the total energy of the wrinkled structure with respect to *A*.

(12) $$\frac{\partial U_{\mathrm{total}}}{\partial A}=\frac{1}{8}{\bar{E}}_{f}h_{f}k^{4}A^{3}+\frac{1}{2}{\bar{E}}_{f}h_{f}k^{2}A\left(f-\varepsilon \right)=0,$$

the following relationship is obtained:

(13) $$A=\frac{2}{k}\sqrt{\varepsilon -f}.$$

By substituting Equation (13) into Equation (11) can obtain

(14) $$\begin{array}{cc} U_{\mathrm{total}} & =\frac{1}{2}{\bar{E}}_{f}h_{f}\varepsilon^{2}-\frac{1}{2}{\bar{E}}_{f}h_{f}{\left(\varepsilon -f\right)}^{2}\\ & \;+\frac{1}{2}{\bar{E}}_{s}\int_{0}^{H}{\left[\varepsilon_{\mathrm{pre}}-\varepsilon +\varepsilon_{\mathrm{applied}}\cos(2\pi \omega t)-\kappa \left(H-y\right)\right]}^{2}dy \end{array}.$$

It can be observed from Equation (11) that when *f* > *ε*, it is evident that *A* = 0 enables *U*_total_ to reach its minimum value. The film is flat. It is only necessary to consider the case where *f* < *ε*. Under this circumstance, it can be readily observed from Equation (14) that *U*_total_ is a monotonic function of *f*. So, minimizing *U*_total_ in Equation (14) with respect to the wavenumber *k* is equal to minimizing *f* with respect to the wavenumber *k*. Through Equation (10), it is only required to determine the value of *k* when *f* reaches its minimum value, denoted as *f*_min_. At this point,

(15) $$A=\frac{2}{k}\sqrt{\varepsilon -f_{\min}}.$$

It is specified that the principle of energy minimization is applied to *ε* and *κ*.

(16) $$\left\{\begin{array}{ll} \frac{\partial U_{\mathrm{total}}}{\partial \varepsilon } & ={\bar{E}}_{f}h_{f}f_{\min}-{\bar{E}}_{s}H(\varepsilon_{\mathrm{pre}}+\varepsilon_{\mathrm{applied}}\cos(2\pi \omega t))+{\bar{E}}_{s}H\varepsilon +\frac{1}{2}\kappa H^{2}{\bar{E}}_{s}=0\\ \frac{\partial U_{\mathrm{total}}}{\partial \kappa } & =\frac{H{\bar{E}}_{s}}{6}\left(3H\varepsilon -3H(\varepsilon_{\mathrm{pre}}+\varepsilon_{\mathrm{applied}}\cos(2\pi \omega t))+2\kappa H^{2}\right)=0 \end{array}\right..$$

Then, the detailed solution process is put in Appendix A.

(17) $$\varepsilon =(\varepsilon_{\mathrm{pre}}+\varepsilon_{\mathrm{applied}}\cos(2\pi \omega t))-\frac{4{\bar{E}}_{f}h_{f}f_{\min}}{{\bar{E}}_{s}H},\;\kappa =\frac{6{\bar{E}}_{f}h_{f}f_{\min}}{{\bar{E}}_{s}H^{2}}.$$

It can be easily derived that the kinetic energy of the thin film/substrate structure, denoted as *E*, can be expressed as

(18) $$E=\frac{1}{2}\rho_{f}\dot{A}{\left(t\right)}^{2},$$

where *ρ*_f_ denotes the density of the thin film.

It can be derived from the Lagrange equation and the Euler function that

(19) $$\frac{d}{dt}\left(\frac{\partial L}{\partial \dot{A}}\right)-\frac{\partial L}{\partial A}=0,$$

where $L=E+U_{\mathrm{total}}$.

The Duffing equation for undamped free vibration is obtained.

(20) $$\rho_{f}\ddot{A}(t)-\frac{1}{8}{\bar{E}}_{f}h_{f}k^{4}A^{3}-\frac{1}{2}{\bar{E}}_{f}h_{f}kA(f-\varepsilon )=0.$$

Dimensionless treatment is performed on Equation (20), with $\bar{A}=\frac{A}{h_{f}},\tau =th_{f}\sqrt{\frac{\alpha_{3}}{\alpha_{1}}}$. The detailed derivation process is placed in Appendix A.

(21) $$\frac{d^{2}\bar{A}}{d\tau^{2}}+\bar{c}\frac{d\bar{A}}{d\tau }+\frac{\alpha_{2}}{{h_{f}}^{2}\alpha_{3}}\bar{A}+{\bar{A}}^{3}=0,$$

where $\bar{c}$ is defined as the dimensionless damping coefficient, and it is usually set at 0.01 in Ou et al. [37].

It is easy to obtain the potential energy function

(22) $$\bar{W}=\frac{\alpha_{2}}{2{h_{f}}^{2}\alpha_{3}}{\bar{A}}^{2}+\frac{{\bar{A}}^{4}}{4}.$$

$\alpha_{2}=-\frac{1}{2}{\bar{E}}_{f}h_{f}k^{2}\left[\frac{h_{f}^{2}k^{2}}{12}+\frac{{\bar{E}}_{s}g\left(kH\right)}{{\bar{E}}_{f}kh_{f}}-\left((\varepsilon_{\mathrm{pre}}+\varepsilon_{\mathrm{applied}}\cos(\bar{\omega }\tau ))-\frac{4{\bar{E}}_{f}h_{f}f_{\min}}{{\bar{E}}_{s}H}\right)\right]$, $\alpha_{3}=-\frac{1}{8}{\bar{E}}_{f}h_{f}k^{4}$ and $\bar{\omega }$ is defined as the dimensionless frequency of the external excitation strain.

## 3. Results and Discussion

In this section, numerical simulations of Equation (21) are performed via the fourth-order Runge–Kutta method. It is aimed to preliminarily analyze the variations in the dynamic behaviors of the thin film under different parameter configurations. Furthermore, bifurcation diagrams of Equation (21) corresponding to the frequency of external excitation, pre-strain, amplitude of strain induced by external excitation, and Young’s modulus of the substrate are constructed respectively. The influences of the different parameters on the critical value at which chaos occurs are explored. The following parameters are defined as Bi et al. [28] and Watanabe et al. [38] $E_{f}=70\mathrm{GPa}$, $v_{f}=0.42$, $E_{s}=2\mathrm{MPa}$, $v_{s}=0.49$, H=0.236mm, $h_{f}=100\mathrm{nm}$.

### 3.1. Comparison with Experiment (FE)

**Figure 2:**
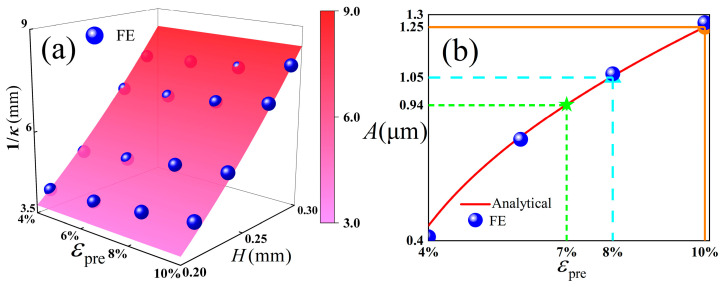
(**a**) The curvature as a function of pre-strain and the thickness of the substrate; (**b**) amplitude of the wrinkled structure as a function of pre-strain.

In order to ensure the accuracy of the theoretical derivation, this study is verified by the finite element method, and the comparison is shown in Figure 2.

It is easy to see from the results of Figure 2 that the finite element method is well compared and verified. The greater the thickness of the substrate, the smaller the curvature of the corrugated structure. With the increase in pre-strain, the value of *A* also increases.

### 3.2. The Effect of the Young’s Moduli of the Substrate

**Figure 3:**
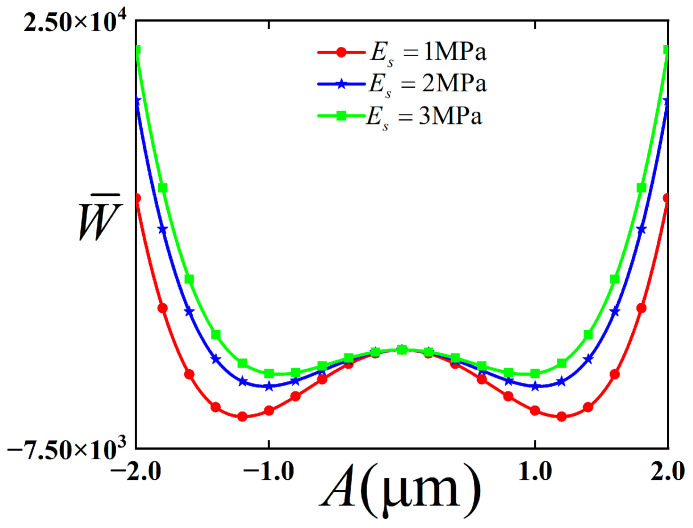
Potential energy function of buckling modal structure with bending configuration with changes of Young’s Modulus of substrate.

The potential energy function of the buckling mode in the bending configuration was studied. The influence of Young’s modulus of the substrate on this potential energy function is discussed, and the result is shown in Figure 3.

As can be seen from Figure 3, with the increase in Young’s modulus of the substrate, the position of the stable equilibrium point approaches the initial dimensionless displacement, and the height of the barrier decreases accordingly. At the same time, the distance between the two stable equilibrium points decreases, and the distance between the wells also decreases. Therefore, the inter-well vibration is more likely to occur.

**Figure 4:**
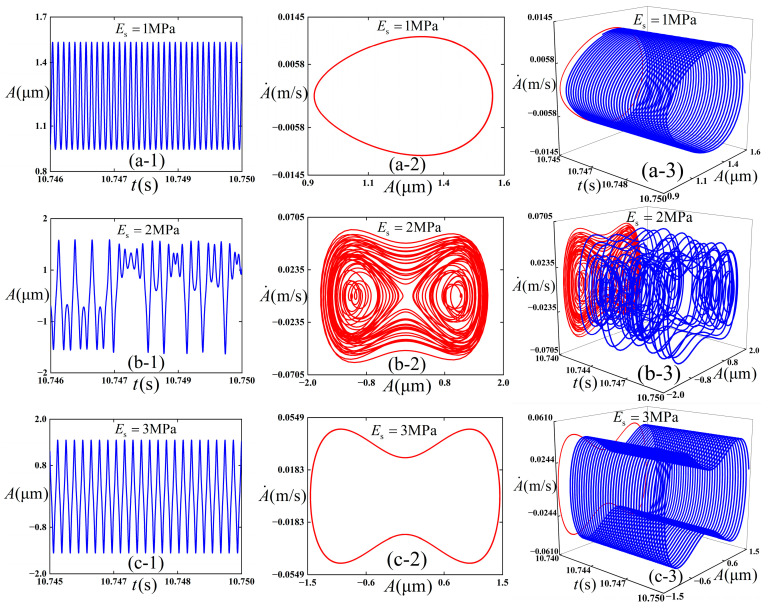
Simulation results of the dynamic response of the thin film/substrate structure. (**a-1**), (**a-2**), and (**a-3**) correspond to the vibration amplitude–time response, phase diagram, and three-dimensional projection diagram when *E*_s_ = 1 MPa, respectively; (**b-1**), (**b-2**), and (**b-3**) are respectively the vibration amplitude–time response, phase diagram, and three-dimensional projection diagram under the condition of *E*_s_ = 2 MPa; and (**c-1**), (**c-2**), and (**c-3**) refer to the vibration amplitude–time response, phase diagram, and three-dimensional projection diagram with *E*_s_ = 3 MPa, respectively.

We intended to preliminarily explore the influence of the selection of Young’s modulus *E*_s_ of the substrate on the dynamic behaviors of the thin film substrate structure in bending configuration, and Figure 4 is plotted accordingly. The following physical parameters ($\bar{c}=0.01$, $\varepsilon_{\mathrm{pre}}=0.08$, $\varepsilon_{\mathrm{applied}}=0.01$, and $\bar{\omega }=12$) are adopted, and the initial conditions ($\bar{A}=0.02$ and $\dot{\bar{A}}=0$) are maintained consistently in the numerical simulations. Total integration time is 50,000, and the step size is 0.01. This paper chooses the integration time 49,960–50,000 in numerical simulation to avoid the wrong analysis caused by the previous instability.

It can be observed from Figure 4 that when the Young’s modulus of the substrate *E*_s_ = 1 MPa (as shown in Figure 4(a-1–a-3)), the amplitude of the thin film in the working state undergoes a stable period-1 state. Combined with Figure 3, because Young’s modulus is small and the well depth is high, the energy is not enough to cross the potential barrier, so the intra-well vibration is done. As the Young’s modulus of the substrate *E*_s_ increases to 2 MPa (as shown in Figure 4(b-1–b-3)), chaotic vibration is exhibited by the amplitude of the thin film. In this case, the cross-well vibration is done. With the further increase in the Young’s modulus of the substrate *E*_s_ to 3 MPa (as shown in Figure 4(c-1–c-3)), a stable period-1 state is retained. Moreover, because the Young’s modulus is large at this time, the well depth is low, and the energy is enough to cross the barrier; therefore, inter-well vibration is done.

**Figure 5:**
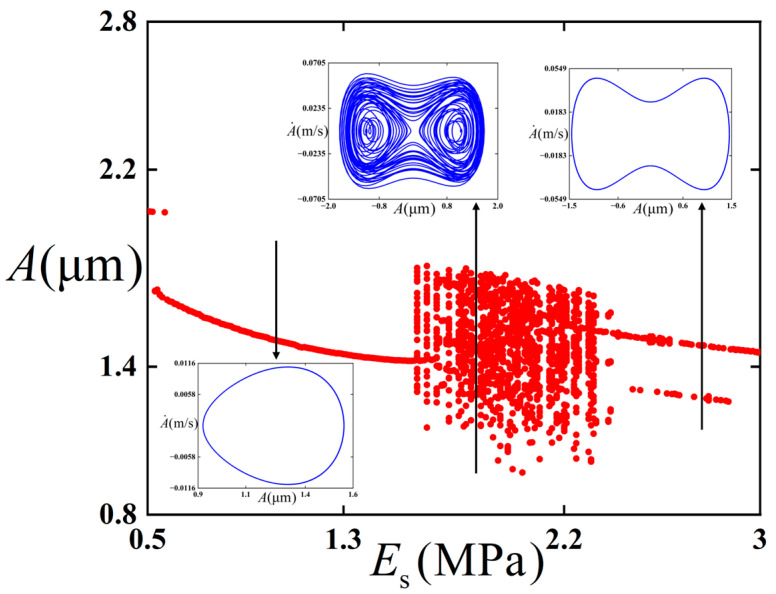
Bifurcation diagram regarding Young’s modulus *E*_s_ of the substrate for the film/substrate structure in the bent state. The red dots represent the bifurcation diagrams, while the blue lines represent the phase diagrams under different parameters.

We intended to further investigate the influence of Young’s modulus *E*_s_ of the substrate on the critical value of the chaotic behavior occurrence. A bifurcation diagram of the thin film/substrate structure in the bent state with respect to Young’s modulus *E*_s_ of the substrate is constructed, as illustrated in Figure 5.

As can be seen from Figure 5, when the Young’s modulus of the substrate increases from 0.5 MPa to 1.59 MPa, a stable period-1 state is maintained. Combined with Figure 3, because the Young’s modulus of the substrate is small and the depth of the potential well is relatively large, the energy is not enough to overcome the potential barrier, so it experiences intra-well vibration during this process. With the increase in Young’s modulus, it enters a chaotic state, which is characterized by aperiodic and irregular displacement response. The structure vibrates in cross-well. When Young’s modulus rises to 2.41 MPa, the chaotic behavior ends. With the further increase in Young’s modulus of the substrate, it reverts to period-vibration. At the same time, the depth of the potential well becomes smaller, which makes the energy enough to overcome the potential barrier, so the structure experiences inter-well vibration.

### 3.3. The Effect of the Pre-Strain

**Figure 6:**
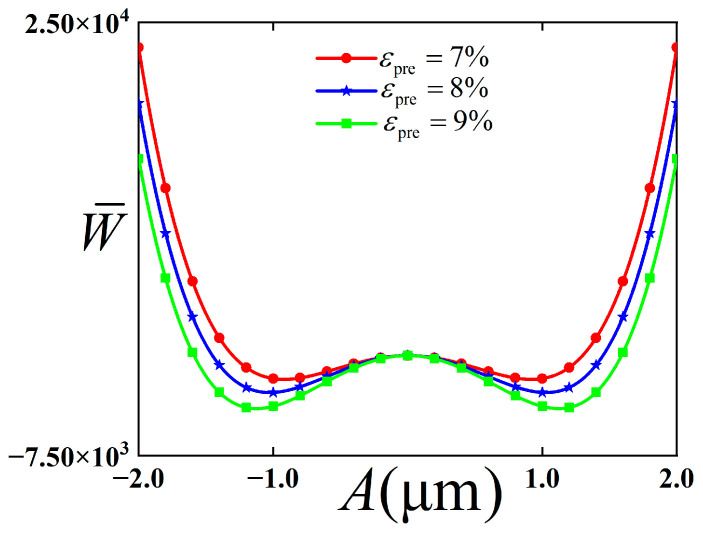
Potential energy function of buckling modal structure with bending configuration with changes of pre-strain of substrate.

To explain the dynamic energy characteristics of the buckling structure, the influence of pre-strain of the substrate on this potential energy function is discussed, and the result is shown in Figure 6.

As can be seen from Figure 6, with the increase in pre-strain, the position of the stable equilibrium point is far from the initial displacement, and the height of the barrier also increases accordingly. At the same time, the distance between the two stable equilibrium points increases, and the distance between the wells also increases. Therefore, the energy required for the structure to vibrate between wells increases, and the intra-well vibration is more likely to occur.

**Figure 7:**
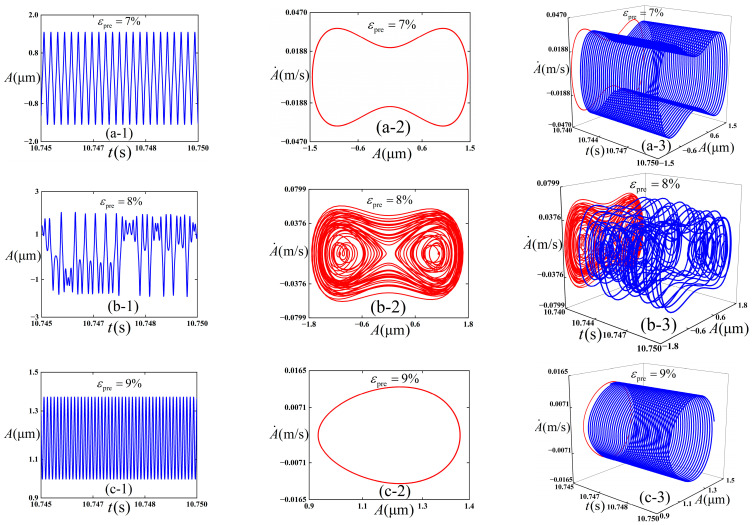
Simulation results of the dynamic response of the thin film/substrate structure. (**a-1**), (**a-2**), and (**a-3**) are respectively the vibration amplitude -time response, phase diagram, and 3D projection diagram when *ε*_pre_ = 0.07; (**b-1**), (**b-2**), and (**b-3**) are respectively the vibration amplitude -time response, phase diagram, and 3D projection diagram when *ε*_pre_ = 0.08; and (**c-1**), (**c-2**), and (**c-3**) are respectively the vibration amplitude -time response, phase diagram, and 3D projection diagram when *ε*_pre_ = 0.09.

We intended to preliminarily explore the influence of the selection of pre-strain *ε*_pre_ on the dynamic behaviors of the thin film/substrate structure, and Figure 7 is plotted. The following physical parameters ($\bar{c}=0.01$, $\varepsilon_{\mathrm{pre}}=0.08$, $\varepsilon_{\mathrm{applied}}=0.01$, and $\bar{\omega }=12$) are adopted, and the initial conditions ($\bar{A}=0.02$ and $\dot{\bar{A}}=0$) are maintained consistently throughout the numerical simulations. Total integration time is 50,000, the step size is 0.01, and choose the integration time 49,960–50,000 in numerical simulation to avoid the wrong analysis caused by the previous instability.

It can be observed from Figure 7 that three types of vibration modes are presented. When the pre-strain *ε*_pre_ of the governing equation is 0.07 (as shown in Figure 7(a-1–a-3)), the amplitude of the thin film in the working state undergoes inter-well vibration, and a stable period-1 state is maintained, as demonstrated in Figure 7(a-2). This is because in this case, the pre-strain is small, the well depth is low, and the energy easily crosses the barrier. When the pre-strain *ε*_pre_ of the substrate increases to 0.08 (as shown in Figure 7(b-1–b-3)), chaotic vibration is exhibited by the amplitude of the thin film. In this case, it can vibrate in cross-well. As the pre-strain *ε*_pre_ of the substrate further increases to 0.09 (as shown in Figure 7(c-1–c-3)), a stable period-1 state is retained. Combined with Figure 6, due to the large pre-strain and high well depth at this time, the energy is not easy to cross the potential barrier, so the intra-well vibration is done.

**Figure 8:**
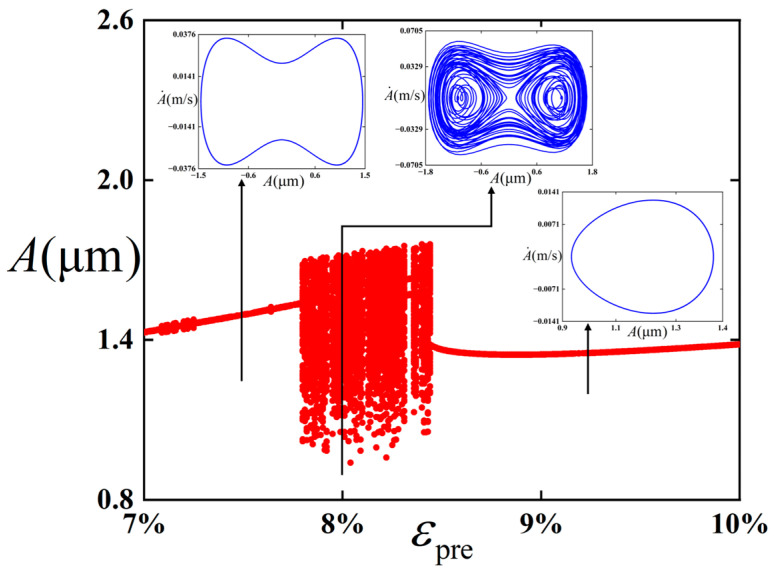
Bifurcation diagram concerning the pre-strain *ε*_pre_ for the film/substrate structure in the bent state. The red dots represent the bifurcation diagrams, while the blue lines represent the phase diagrams under different parameters.

We intended to further explore the influence of the pre-strain *ε*_pre_ on the critical value of the chaotic behavior occurrence, and Figure 8 is plotted accordingly.

It can be easily observed from Figure 8 that when the value of pre-strain *ε*_pre_ is within the range of 0.07 to 0.1, the bifurcation diagram can be divided into three regions. When the pre-strain *ε*_pre_ ranges from 0.07 to 0.0781, a stable period-1 state is maintained. And combined with Figure 6, in this process, the pre-strain is small, the well depth is low, and the energy is easy to cross the potential barrier and do inter-well vibration. As the pre-strain *ε*_pre_ further increases from 0.0782 to 0.0845, chaotic vibration is experienced, which is characterized by aperiodic and irregular displacement response. The structure vibrates in cross-well. When the pre-strain *ε*_pre_ continues to increase to 0.1, during this process, it recovers to a stable period-1 state. And in this process, the pre-strain is relatively large, the well depth is high, and the energy is not enough to cross the potential barrier and do intra-well vibration.

### 3.4. The Effect of the Frequency

**Figure 9:**
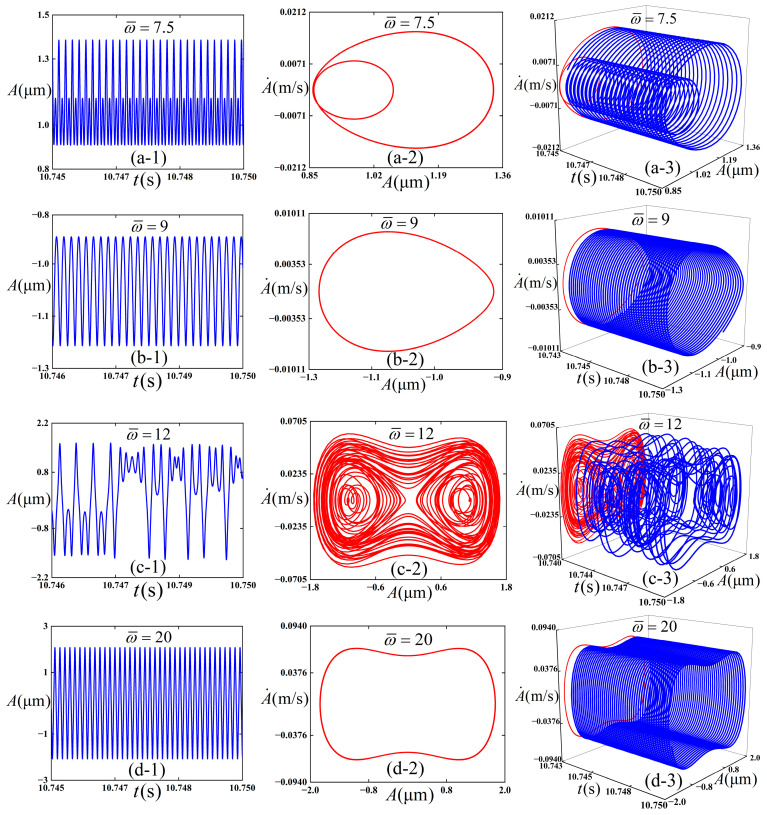
Simulation results of the dynamic response of the thin film/substrate structure. (**a-1**), (**a-2**), and (**a-3**) are respectively vibration amplitude -time response, phase diagram, and 3D projection diagram when $\bar{\omega }$ = 7.5; (**b-1**), (**b-2**), and (**b-3**) are respectively the vibration amplitude–time response, phase diagram, and 3D projection diagram when $\bar{\omega }$ = 9; and (**c-1**), (**c-2**), and (**c-3**) are respectively the vibration amplitude -time response, phase diagram, and 3D projection diagram when $\bar{\omega }$ = 12; (**d-1**), (**d-2**), and (**d-3**) are respectively the vibration amplitude -time response, phase diagram, and 3D projection diagram when $\bar{\omega }$ = 20.

We intended to preliminarily explore the influence of the variation in frequency on the dynamic behaviors of the thin film/substrate structure, and Figure 9 is plotted. The following physical parameters (*c* = 0.01, *ε*_pre_ = 0.08, and *ε*_applied_ = 0.01) are adopted, and the initial conditions ($\bar{A}=0.02$ and $\dot{\bar{A}}=0$) are consistently maintained throughout the numerical simulations. Total integration time is 50,000, and the step size is 0.01.

It can be observed from Figure 9 that four types of vibration modes are presented therein. When the $\bar{\omega }$ of the pre-strain under external excitation is 7.5 (as shown in Figure 9(a1–a3)), the structure undergoes intra-well vibration, and a stable period-2 state is exhibited. When the $\bar{\omega }$ under external excitation increases to 9 (as shown in Figure 9(b1–b3)), it still undergoes intra-well vibration but transforms into a stable period-1 state. As the $\bar{\omega }$ of the external excitation further increases to 12 (as shown in Figure 9(c1–c3)), it experiences chaotic vibration, which is characterized by aperiodic and irregular displacement response. The structure vibrates in cross-well. When $\bar{\omega }$ of the external excitation increases to 20 (as shown in Figure 9(d1–d3)), it performs inter-well vibration and recovers to a stable period-1 vibration.

**Figure 10:**
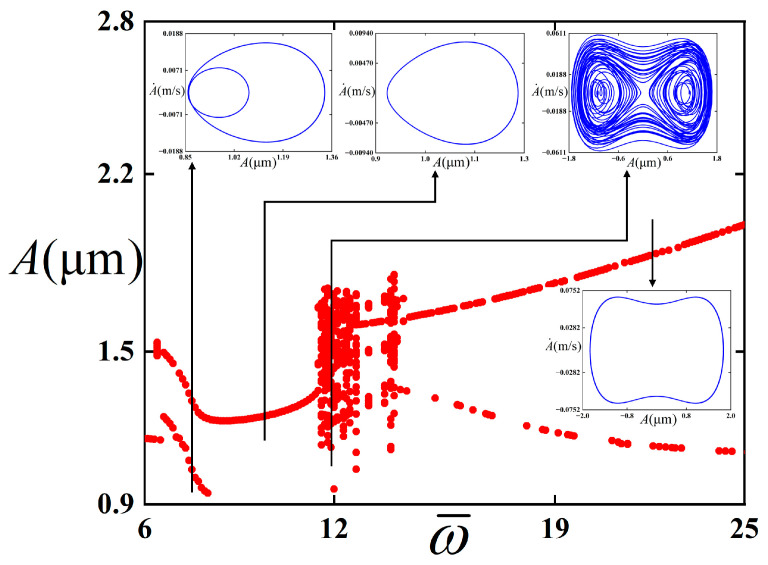
Bifurcation diagram of the $\bar{\omega }$ for the film/substrate structure in the bent state under periodic excitation. The red dots represent the bifurcation diagrams, while the blue lines represent the phase diagrams under different parameters.

We intended to further investigate the influence of the $\bar{\omega }$ of the external periodic excitation on the stability of the thin film/substrate structure. A bifurcation diagram of the thin film/substrate structure in the bent state with respect to $\bar{\omega }$ of the periodic excitation is constructed in this study, as illustrated in Figure 10.

It can be observed from Figure 10 that the structure exhibits more abundant dynamic behaviors with the variation in the $\bar{\omega }$. It is adopted in this study that $\bar{\omega }$ is in the range of 6 to 25. When $\bar{\omega }$ is between 6 and 6.5, a period-1 steady state is maintained. As $\bar{\omega }$ increases continuously from 6.6, the vibration behavior of the film amplitude during operation gradually manifests a period-2 steady state until $\bar{\omega }$ = 8, where the period-2 state terminates. In this case, the intra-well vibration was maintained. With $\bar{\omega }$ further increasing to 11.5, the vibration behavior reverts to the period-1 steady state. When $\bar{\omega }$ is in the interval of 11.6 to 13.9, it undergoes chaos, which is characterized by aperiodic and irregular displacement response. The structure vibrates in cross-well. With the continuous increase in $\bar{\omega }$, the vibration resumes the period-1 steady state, and in this process, intra-well vibration and inter-well vibration appear successively.

### 3.5. The Effect of the Excitation Amplitude

**Figure 11:**
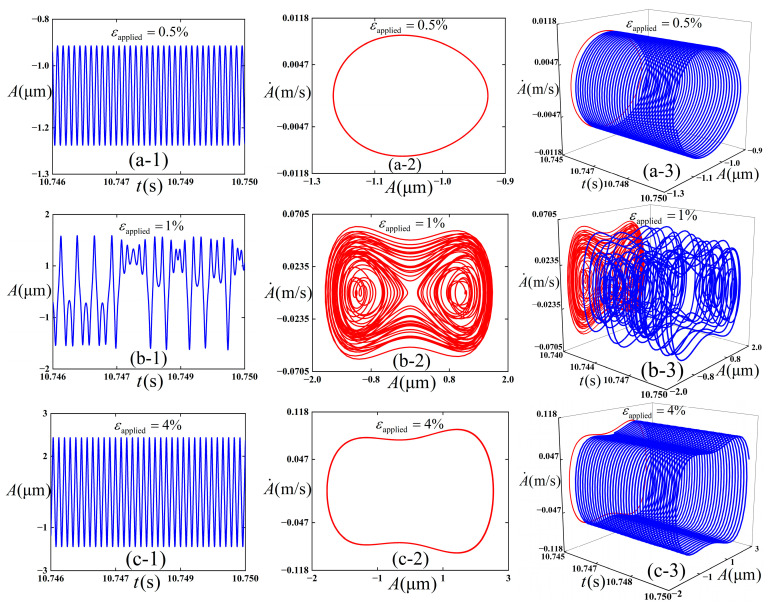
Simulation results of the dynamic response of the thin film/substrate structure. (**a-1**), (**a-2**), and (**a-3**) are respectively the vibration amplitude -time response, phase diagram, and 3D projection diagram when *ε*_applied_ = 0.005; (**b-1**), (**b-2**), and (**b-3**) are respectively the vibration amplitude -time response, phase diagram, and 3D projection diagram when *ε*_applied_ = 0.01; and (**c-1**), (**c-2**), and (**c-3**) are respectively the vibration amplitude -time response, phase diagram, and 3D projection diagram when *ε*_applied_ = 0.04.

We intended to preliminarily explore the influence of the selection of excitation amplitude *ε*_applied_ on the dynamic behaviors of the thin film/substrate structure, and Figure 11 is plotted accordingly. In Figure 11, the response diagrams of Equation (21) under different values of *ε*_applied_ are calculated by virtue of the fourth-order Runge–Kutta method. The following physical parameters ($\bar{c}=0.01$, *ε*_pre_ = 0.08, and $\bar{\omega }=12$) are adopted, and the initial conditions ($\bar{A}=0.02$ and $\dot{\bar{A}}=0$) are consistently maintained in the numerical simulations. Total integration time is 50,000, and the step size is 0.01.

It can be observed from Figure 11 that three types of vibration modes are presented therein. When *ε*_applied_ is set to 0.005, a stable period-1 vibration state is maintained, which performs intra-well vibration, and the vibration amplitude of the film during vibration is relatively small. When *ε*_applied_ is set to 0.01, chaotic vibration is experienced, which is characterized by aperiodic and irregular displacement response. The structure vibrates in cross-well. When *ε*_applied_ is set to 0.04, it recovers to a stable period-1 vibration state and undergoes inter-well vibration in an asymmetric double potential well on the left and right; at this point, the vibration amplitude of the film is relatively high.

**Figure 12:**
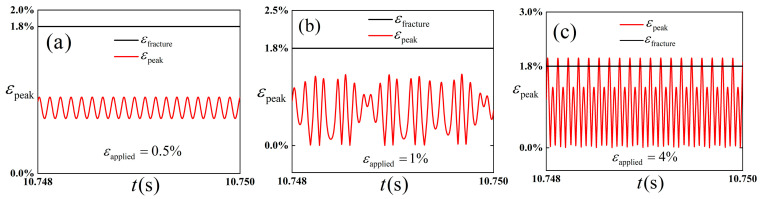
Peak strain under different excitation amplitudes. (**a**) Peak strain when the amplitude of excitation strain is 0.5%. (**b**) Peak strain when the amplitude of excitation strain is 1%. (**c**) Peak strain when the amplitude of excitation strain is 4%.

In order to study the influence of the amplitude of external excitation strain on the peak strain of thin films and predict whether fracture occurs, Figure 12 is plotted.

Figure 12 shows the peak strain of the film when the amplitude of external excitation strain is 0.5%, 1%, and 4%, respectively. Among them, the fracture strain is 1.8% according to Bi et al. [39]. It is easy to see from the figure that when the amplitude of excitation strain is 0.5%, combined with Figure 11, the structure undergoes intra-well vibration, and the peak strain is lower than the fracture strain, so no fracture occurs. When the excitation strain amplitude is 1%, although the structure appears chaotic, the peak strain is lower than the fracture strain because of the small excitation strain. When the amplitude of excitation strain continues to increase, as shown in Figure 11, inter-well vibration occurs, and the peak strain is higher than the fracture strain at some moments.

**Figure 13:**
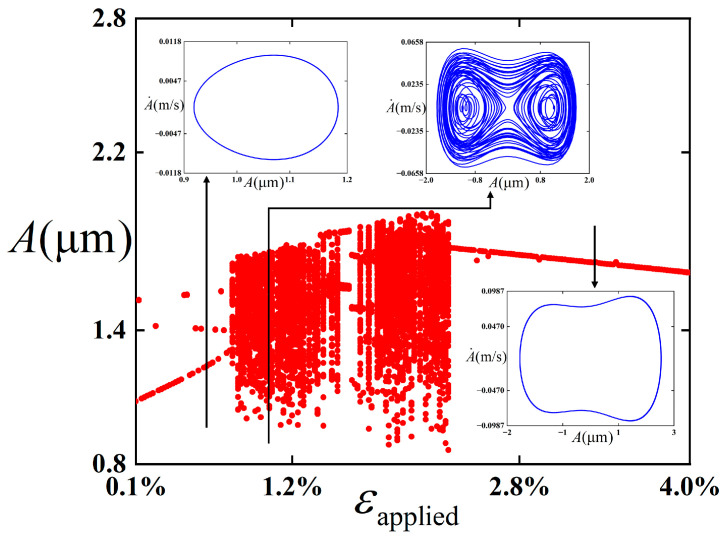
Bifurcation diagrams of the film/substrate structure under the bending state with respect to the amplitude *ε*_applied_ of periodic excitation. The red dots represent the bifurcation diagrams, while the blue lines represent the phase diagrams under different parameters.

To investigate the influence of the amplitude *ε*_applied_ of periodically excited strain on the stability for the film/substrate structure under the bending state, bifurcation diagrams of the structure with respect to the amplitude *ε*_applied_ of periodic excitation are plotted in this study (as shown in Figure 13). The following physical parameters ($\bar{c}=0.01$, *ε*_pre_ = 0.08, and $\bar{\omega }=12$) are selected.

It can be observed from Figure 13 that the influence of *ε*_applied_ in the range of 0 to 0.04 on the dynamic behaviors of the structure is examined in this study. When *ε*_applied_ is between 0 and 0.0074, the period-1 vibration is maintained; during this process, the vibration form is mainly intra-well. With *ε*_applied_ further increasing from 0.0075 to 0.0234, the structure undergoes chaotic vibration, where a short-lived multi-period vibration is experienced. Subsequently, as *ε*_applied_ increases to 0.04, the vibration maintains a period-1 steady state and performs inter-well vibration.

It can be preliminarily concluded from the analysis of Figure 11, Figure 12 and Figure 13 that the selection of amplitude of the external excitation exerts an influence on the dynamic response. Specifically, when the amplitude of excitation strain is small, intra-well vibration occurs, the peak strain is lower than the fracture strain, and no fracture occurs. With the increase in the amplitude of excitation strain, the structure gradually appears chaotic, which is characterized by aperiodic and irregular displacement response. The structure vibrates in cross-well. When the amplitude of excitation strain continues to increase, inter-well vibration occurs, and the peak strain is higher than the fracture strain at some moments.

### 3.6. The Effect of the Substrate Thickness

**Figure 14:**
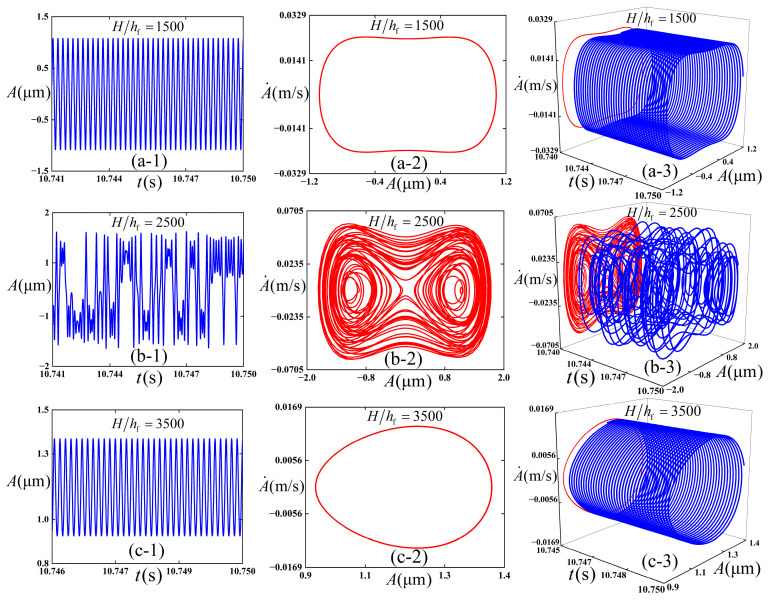
Simulation results of the dynamic response of the thin film/substrate structure. (**a-1**), (**a-2**), and (**a-3**) are respectively the vibration amplitude -time response, phase diagram, and 3D projection diagram when $H/h_{f}$ = 1500; (**b-1**), (**b-2**), and (**b-3**) are respectively the vibration amplitude -time response, phase diagram, and 3D projection diagram when $H/h_{f}$ = 2500; and (**c-1**), (**c-2**), and (**c-3**) are respectively the vibration amplitude -time response, phase diagram, and 3D projection diagram when $H/h_{f}$ = 3500.

We intended to preliminarily explore the influence of the selection of substrate thickness on the dynamic behaviors of the thin film/substrate structure, and Figure 14 is plotted accordingly. The following physical parameters ($\bar{c}=0.01$, *ε*_pre_ = 0.08, *ε*_applied_ = 0.01, and $\bar{\omega }=12$) are adopted, and the initial conditions ($\bar{A}=0.02$ and $\dot{\bar{A}}=0$) are maintained consistently throughout the numerical simulations. Total integration time is 50,000, and the step size is 0.01.

It is easy to see from Figure 14 that when the thickness of the substrate is 1500 times that of the thin film, the film vibrates stably for period 1, and the inter-well vibration occurs. When the thickness of the substrate is 2500 times that of the thin film, the thin film experiences chaotic vibration, which is characterized by aperiodic and irregular displacement response. The structure vibrates in cross-well. When the thickness of the substrate is 3500 times that of the thin film, the structure returns to a steady state, and the intra-well vibration occurs.

**Figure 15:**
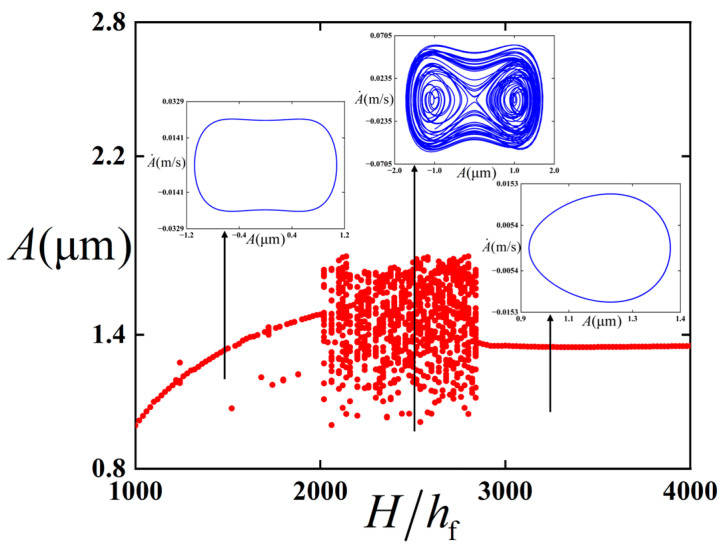
Bifurcation diagram of thin film/substrate structure with respect to substrate thickness in bending configuration. The red dots represent the bifurcation diagrams, while the blue lines represent the phase diagrams under different parameters.

For further discussion about the dynamic influence of the change in substrate thickness, the bifurcation diagram of the thin film/substrate structure with respect to substrate thickness (as shown in Figure 15) is plotted.

It is easy to see from Figure 15 that when the thickness of the substrate is small relative to the thickness of the film, with the increase in the thickness of the substrate, the dynamic behavior of the structure will change, such as from period 1 vibration to chaos. However, when the thickness of the substrate is large enough relative to the thickness of the thin film, the stability of the structure, the vibration mode, and the vibration amplitude of the thin film will not change obviously, even if the thickness of the substrate continues to increase to an infinite thickness substrate under the classical assumption.

It should be noted that PDMS is inherently viscoelastic, exhibiting rate-dependent behavior and energy dissipation under cyclic loading. This simplified model is primarily applicable to small strain ranges in this paper. For applications involving high-frequency vibrations, long-term creep, or stress relaxation, viscoelastic constitutive models such as the Standard Linear Solid model, Maxwell model, or Kelvin-Voigt model are recommended, which will be pursued in future work.

## 4. Conclusions

Based on the principle of minimum energy and the method of Lagrange function, the dynamic buckling response of the film/substrate structure under the bending state during operation is investigated in this study. Dynamic equations are established, and the fourth-order Runge–Kutta method is employed to perform numerical simulations on the dynamic response of the film/substrate structure. A preliminary analysis is conducted on the variations in the dynamic behaviors caused by different values of the substrate Young’s modulus *E*_s_, pre-strain *ε*_pre_, dimensionless frequency $\bar{\omega }$ of periodic excitation, and excitation amplitude *ε*_applied_, respectively. Bifurcation diagrams of the thin film/substrate structure under each parameter are plotted, and an exploration is carried out on the influence of *E*_s_, *ε*_pre_, $\bar{\omega }$, *ε*_applied_, and *H*/*h*_f_ on the critical values of the occurrence of chaotic behaviors. Finally, the following conclusions can be drawn from this study:

1. It is generally observed that the transition between intra-well vibration and inter-well vibration in the thin-film vibration structure is accompanied by chaotic behavior.
2. With the increase in pre-strain, the distance between two stable equilibrium points of the potential energy function increases, and the distance between wells and the height of the potential barrier also increase accordingly. However, the influence of Young’s modulus of substrate on the potential energy function shows the opposite result.
3. It is found that the substrate Young’s modulus *E*_s_, pre-strain *ε*_pre_, and excitation amplitude *ε*_applied_ of the structure all exert an influence on the stability. And the $\bar{\omega }$ of periodic excitation will cause a period 2 vibration.
4. It is revealed that differences in the values of the substrate Young’s modulus *E*_s_, pre-strain *ε*_pre_, $\bar{\omega }$ of periodic excitation, and excitation amplitude *ε*_applied_ can result in variations in the vibration forms of the structure, including intra-well vibration and inter-well vibration.

It is anticipated that this study will provide novel insights for the design of bending sensors based on the film/substrate structure. By regulating the physical parameters, chaos can be effectively avoided, and the robustness of the bending sensor in working conditions can be guaranteed.

## Data Availability

The original contributions presented in this study are included in the article. Further inquiries can be directed to the corresponding author.

## Nomenclature

*h*_f_	The thickness of the film
*H*	The thickness of the substrate
*v*_f_	Poisson’s ratios of the film
*v_s_*	Poisson’s ratios of the substrate
${\bar{E}}_{f}$	The equivalent Young’s modulus of the film
${\bar{E}}_{s}$	The equivalent Young’s modulus of the substrate
*u*	The axial displacement function
*ε_x_*	The central axial strain of the thin film
*ε*	The strain of the thin film
*w*	The lateral displacement of the thin film
*t*	Time
*A*(*t*)	The time-varying wrinkle amplitude of this structure
*k*	The characteristic wave number
*U*_b_	The bending energy of the film
*U*_m_	The membrane energy of the film
*A*	The abbreviation of *A*(*t*)
*U*_s_	The strain energy of the substrate
*U*_total_	The total energy
$\dot{A}\left(t\right)$	The first derivative of *A*(*t*) with respect to *t*
$\ddot{A}\left(t\right)$	The second derivative of *A*(*t*) with respect to *t*
*E*	The total kinetic energy of the film/substrate structure
*ρ*_f_	The density of the film
*ε*_applied_	The amplitude of the external excitation strain
*ε*_pre_	The pre-strain
*κ*	The curvature of the substrate
*c*	The damping term
*ω*	The frequency of the external excitation
$\bar{\omega }$	The dimensionless frequency of external excitation
*τ*	The dimensionless time
$\bar{c}$	The dimensionless damping coefficient
$\bar{W}$	The potential energy function
*ε*_fracture_	The fracture strain
*ε*_peak_	The peak strain

## Appendix A

In this section, we give the solution process of Formula (16) to Formula (17) and the process of Formula (20) to Formula (21).

1. The solution process of Equation (16) to Equation (17)

(A1) $$\left\{\begin{array}{l} \frac{\partial U_{\mathrm{total}}}{\partial \varepsilon }={\bar{E}}_{f}h_{f}f_{\min}-{\bar{E}}_{s}H(\varepsilon_{\mathrm{pre}}+\varepsilon_{\mathrm{applied}}\cos(2\pi \omega t))+{\bar{E}}_{s}H\varepsilon +\frac{1}{2}\kappa H^{2}{\bar{E}}_{s}=0\\ \frac{\partial U_{\mathrm{total}}}{\partial \kappa }=\frac{H{\bar{E}}_{s}}{6}\left(3H\varepsilon -3H(\varepsilon_{\mathrm{pre}}+\varepsilon_{\mathrm{applied}}\cos(2\pi \omega t))+2\kappa H^{2}\right)=0 \end{array}\right..$$

(A2) $$\left(3H\varepsilon -3H(\varepsilon_{\mathrm{pre}}+\varepsilon_{\mathrm{applied}}\cos(2\pi \omega t))+2\kappa H^{2}\right)=0$$

and

(A3) $${\bar{E}}_{f}h_{f}f_{\min}-{\bar{E}}_{s}H(\varepsilon_{\mathrm{pre}}+\varepsilon_{\mathrm{applied}}\cos(2\pi \omega t))+{\bar{E}}_{s}H\varepsilon +\frac{1}{2}\kappa H^{2}{\bar{E}}_{s}=0$$

According to Formula (A2)

(A4) $$\varepsilon =(\varepsilon_{\mathrm{pre}}+\varepsilon_{\mathrm{applied}}\cos(2\pi \omega t))-\frac{2}{3}\kappa H$$

Substitute (A4) into (A3) to obtain

(A5) $${\bar{E}}_{f}h_{f}f_{\min}-\frac{1}{6}\kappa H^{2}{\bar{E}}_{s}=0$$

Then

(A6) $$\kappa =\frac{6{\bar{E}}_{f}h_{f}f_{\min}}{H^{2}{\bar{E}}_{s}}$$

Substitute (A6) into (A4) to obtain

(A7) $$\varepsilon =(\varepsilon_{\mathrm{pre}}+\varepsilon_{\mathrm{applied}}\cos(2\pi \omega t))-\frac{4{\bar{E}}_{f}h_{f}f_{\min}}{H{\bar{E}}_{s}}$$

2. The solution process of Equation (20) to Equation (21)

It can be obtained by substituting Equations (10) and (17) into Equation (20) with the damping term incorporated that

(A8) $$\alpha_{1}\ddot{A}(t)+c\dot{A}(t)+\alpha_{2}A(t)+\alpha_{3}A{\left(t\right)}^{3}=0,$$

where *c* is defined as the damping coefficient.

$$\begin{array}{l} \alpha_{1}=\rho_{f},\\ \alpha_{2}=-\frac{1}{2}{\bar{E}}_{f}h_{f}k^{2}\left[\frac{h_{f}^{2}k^{2}}{12}+\frac{{\bar{E}}_{s}g\left(kH\right)}{{\bar{E}}_{f}kh_{f}}-\left((\varepsilon_{\mathrm{pre}}+\varepsilon_{\mathrm{applied}}\cos(2\pi \omega t))-\frac{4{\bar{E}}_{f}h_{f}f_{\min}}{{\bar{E}}_{s}H}\right)\right],\\ \alpha_{3}=-\frac{1}{8}{\bar{E}}_{f}h_{f}k^{4}. \end{array}$$

Dimensionless treatment is performed on Equation (A8), with $\bar{A}=\frac{A}{h_{f}},\tau =th_{f}\sqrt{\frac{\alpha_{3}}{\alpha_{1}}}$.

(A9) $$\begin{array}{l} \frac{d\bar{A}}{d\tau }=\frac{d\bar{A}}{dA}\times \frac{dA}{dt}\times \frac{dt}{d\tau }=\frac{1}{{h_{f}}^{2}}\times \sqrt{\frac{\alpha_{1}}{\alpha_{3}}}\times \frac{dA}{dt}\\ \frac{d^{2}\bar{A}}{d\tau^{2}}=\frac{\frac{d\bar{A}}{d\tau }}{dt}\times \frac{dt}{d\tau }=\frac{1}{{h_{f}}^{3}}\times \frac{\alpha_{1}}{\alpha_{3}}\times \frac{d^{2}A}{dt^{2}} \end{array},$$

where $\bar{A}$ is defined as the dimensionless amplitude, and *τ* is defined as the dimensionless time. Eventually, the dimensionless system is derived.

(A10) $$\frac{d^{2}\bar{A}}{d\tau^{2}}+\bar{c}\frac{d\bar{A}}{d\tau }+\frac{\alpha_{2}}{{h_{f}}^{2}\alpha_{3}}\bar{A}+{\bar{A}}^{3}=0.$$
